# *Acanthamoeba* Mannose and Laminin Binding Proteins Variation across Species and Genotypes

**DOI:** 10.3390/microorganisms10112162

**Published:** 2022-10-31

**Authors:** Daniele Corsaro

**Affiliations:** CHLAREAS, 54500 Vandoeuvre-lès-Nancy, France; corsaro@gmx.fr

**Keywords:** *Acanthamoeba*, mannose-binding protein, laminin-binding protein, variability, phylogenesis

## Abstract

*Acanthamoeba* is a ubiquitous free-living amoeba capable of being an opportunistic pathogen in humans and animals. A critical step in infection is the adhesion of the amoeba to host cells and tissues, and two major parasite adhesins, mannose-binding protein (MBP) and laminin-binding protein (LBP), are known to recognize the cell surface glycoproteins and those of the extracellular matrix, respectively. In this study, the available genomes of *Acanthamoeba* were analysed to recover the sequences of MBP and LBP using previously published genetic data. Genes for both proteins were successfully obtained from strains belonging to various genotypes (T4A, T4D, T4G, T4F, T2, T5, T10, T22, T7 and T18), resulting in a single gene for LBP but identifying two types of MBP, MBP1 and MBP2. Phylogenetic analysis based on deduced amino acid sequences shows that both MBP and LBP have a branching pattern that is consistent with that based on 18S rDNA, indicating that changes in both proteins occurred during diversification of *Acanthamoeba* lines. Notably, all MBPs possess a conserved motif, shared with some bacterial C-type lectins, which could be the recognition site for mannose binding.

## 1. Introduction

Free-living amoebae of the genus *Acanthamoeba* (Amoebozoa, Discosea, Centramoebida) are ubiquitous worldwide in any type of natural or man-made environment. The active stage, the trophozoite, feeds on bacteria and other microbial organisms, while the dormant cyst is highly resistant to many physical and chemical stresses. *Acanthamoeba* can, however, occasionally infect humans and other animals, causing two main diseases [1]: an often-fatal granulomatous amoebic encephalitis (GAE) which follows invasion of the central nervous system [2], and a sight-threatening amoebic keratitis (AK) due to infection of the surface of the eye [3]. Once inside the host, the amoebae adhere to its cells and trigger a series of cascading reactions involving both the release of parasite proteases and the activation of host cell factors, leading to the destruction of epithelium and extracellular matrix and the progression of invasion [4,5].

**Figure 1:**
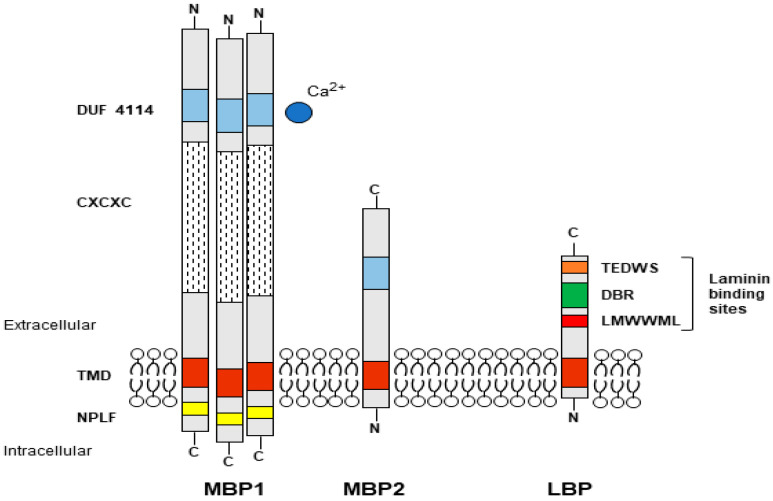
Schematic drawings of *Acanthamoeba* adhesins. Mannose binding protein 1 (MBP1) and 2 (MBP2); Laminin binding protein (LBP). The putative site for mannose binding in both MBP1 and MBP2 should be in the DUF 4114 region and involve a divalent cation. For LBP, the laminin binding sites correspond to the palindromic motif, the direct binding region (DBR), and the TEDWS motif. See text for more details and explanation.

Adhesion is, therefore, a critical step in infection, and one of the main *Acanthamoeba* adhesins identified is the mannose-binding protein (MBP1), a lectin-like glycoprotein located on the surface of trophozoites, which recognizes mannose residues of glycoproteins of host cells [6,7,8]. MBP1 is a protein of approximately 400 kDa composed of several 130 kDa subunits. It has a long extracellular N-terminal part, a transmembrane domain and a short C-terminal part containing the NPLF motif known to participate in intracellular signalling events. The mannose-specific recognition domain is expected to be located in the extracellular part, but it has not yet been identified [7,9]. Another important *Acanthamoeba* adhesin is the laminin-binding protein (LBP), which allows further progression of infected tissues [10,11], as laminin is a major glycoprotein of the extracellular matrix separating epithelia from other tissues. *Acanthamoeba* LBP belongs to the family of non-integrin 37/67-kDa laminin receptors (37/67LR), also involved as receptors for viruses and other pathogens as well as in other cellular processes such as motility and differentiation [12]. LBP homologs are present in all organisms including prokaryotes as this adhesin derives from a 40S ribosomal protein which acquired the ability to bind laminin with evolution [13]. Overall, LBPs have a short transmembrane domain at the N-terminal, and three recognition domains for laminin on the extracellular C-terminal domain, comprising a palindromic LMWWML motif located in the peptide G [14], a direct binding region (DBR), and TEDWS motif repeats (Figure 1).

The sequences of the two adhesins were obtained by cDNA cloning, from a strain attributed to *A. castellanii* for MBP1 [7], and from *A. healyi* for LBP [10], two very distant species, belonging to different morphological groups and genetic lines. Indeed, according to the cyst shapes and the variations in the nuclear small subunit (SSU) of the ribosomal RNA gene (18S rDNA), *A. castellanii* is placed in group 2, genotype T4, while *A. healyi* is in group 3, genotype T12 [15,16,17,18]. Biomolecular studies based on the 18S rDNA revealed high diversity within *Acanthamoeba*, currently comprising at least 23 genotypes, T1-T23 [18,19,20], which partly correspond to the traditional species [21]. In this study the available data are analysed in order to assess if and how MBP and LBP are present and vary in the different lines of *Acanthamoeba*.

## 2. Materials and Methods

*Acanthamoeba* genomes available on the NCBI portal were analysed by BLAST using as query sequences the complete MBP1 gene from the AK strain MEEI 0184 (GenBank ID AY604039) [7] and the complete LBP gene from the environmental strain OC-3A (ATCC 30866) of *A. healyi* (GenBank ID AY351649) [10]. The genomic regions obtained were analysed with Genscan [22] and Augustus [23] to generate coding fragments (exons), and further verified and if necessary corrected, using as guides the known amino acid sequences of MBP1 (GenBank ID AAT37864) and LBP (GenBank ID AAQ63482).

Amino acid sequences were separately aligned with COBALT [24] and phylogenetic trees (1000 bootstraps) were inferred with protein maximum likelihood (ML, JTT + *G*:4 model) using TREEFINDER [25], and distance (Minimum Evolution, ME) and Maximum Parsimony (MP) using MEGA7 [26]. As an outgroup, the sequences hereinafter referred to as MBP2 (see below) were used for MBP, whereas for LBP, the trees were rooted on the sequence of *Balamuthia mandrillaris*, recovered from its genome as described above. Similarity and identity values were estimated with MatGat [27] using Blocks Substitution Matrix 50 (BLOSUM50). Molecular weights were predicted by using BioEdit [28], and putative *N*-glycosylation sites were identified by using NetNGlyc 1.0 [29].

A reference 18S rDNA tree Iding the sequences of the studied strains and representatives of all genotypes was constructed with ML (GTR *G* + I:4; 1000 bootstraps) after MAFFT alignment and manual editing, as previously described [19,30].

## 3. Results

### 3.1. 18S rDNA Phylogeny

**Figure 2:**
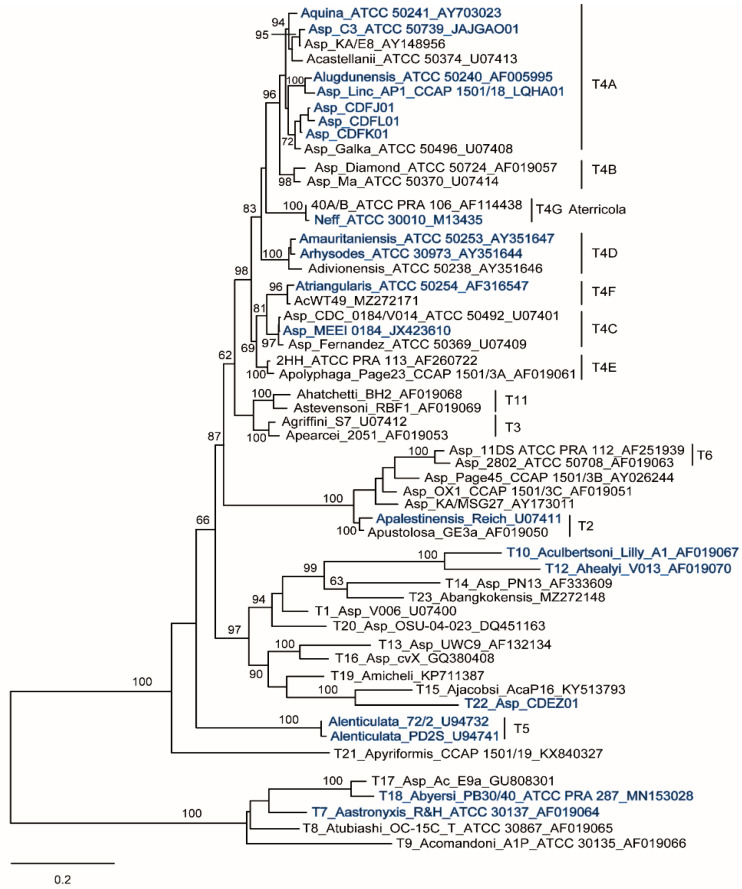
The 18S rDNA phylogeny. Maximum likelihood tree (1000 bootstraps) based on complete 18S rDNA sequences including representatives of all *Acanthamoeba* genotypes, rooted on members of morphological group 1. Strains analysed herein are highlighted in blue.

An 18S tree was constructed including members of all *Acanthamoeba* genotypes as well as the strains analysed here for the genes of the two adhesins (Figure 2).

As expected, the resulting tree is consistent with results obtained in previous studies including a larger number of strains and based on both nuclear and mitochondrial SSU rDNA [21] as well as internal transcribed spacer (ITS) region and the large subunit (LSU) of the nuclear rDNA operon [31]. The analysis places the MEEI 0184 strain in the T4C lineage, which includes various isolates from around the world implicated in AK, probably forming one or more species, but distinct from *A. castellanii* (T4A) and closer to *A. triangularis* (T4F) [21]. For *A. lenticulata* (T5), 18S sequences from only two strains were used here since in the third strain, PT14, the likely presence of a group 1 intron prevented complete sequencing of the gene [31]. It should be noted that for *A. healyi* (T12), the 18S rDNA was from the strain type CDC:1283:V013 (=V013) [17], isolated from a non-fatal case of brain granuloma, while LBP was from the freshwater strain OC-3A [32], deposited in the American Type Culture Collection (ATCC 30866). There is often confusion between these two strains of *A. healyi*, mistakenly assuming that ATCC 30866 is from GAE. Studies including this species but using the ATCC strain have actually analysed the freshwater strain OC-3A. Nuclear and mitochondrial SSU rDNA sequences are available only for V013 [18,33], and their deduced restriction profiles are different, although very similar from those reported for OC-3A [34,35].

### 3.2. Recovery of Mannose and Laminin Binding Protein Genes: General Features

The genes for MBP1 from strain MEEI (T4C) and LBP from *A. healyi* OC-3A (T12) were used to search for respective orthologs in publicly available genomes. The LBP gene was successfully recovered from all analysed genomes including that of sister species *Balamuthia mandrillaris*, while the MBP1 gene appears to be missing not only in *Balamuthia*, but also in group 1 species, *A. astronyxis* (T7) and *A. byersi* (T18). The two genes both differ by their length and their structure (number of exon/intron) giving proteins of different sizes depending on the genotype, and all genes have U2-type GT/AG spliceosomal introns, 1 to 3 for LBP, and 3 to 8 for MBP1, except for the LBP gene of *A. healyi* which codes for a single transcript. Despite these variations, the deduced amino acids give for the two proteins well-conserved overall structures congruent with the literature data (Table 1) (Table S1).

All LBPs present the recognition sites for laminin, including an LMFWLL motif or more rarely LLYWLL (Group 1 species and *Balamuthia*) corresponding to the palindromic LMWWML motif of the peptide G, but a single complete TEDWS element (as TEEWG), and all have at the N-terminus a short sequence of hydrophobic residues corresponding to the transmembrane domain. Notably, as expected, an identical gene for LBP, consisting of three exons and two introns, is present in all three available genomes of *A. terricola* (strain Neff, T4G), but it is annotated as a pseudogene in the AHJI01 genome (ACA1_385450) because the introns are not recognized. Furthermore, the multiple alignment of LBPs suggests that the C-terminus of the original sequence of *A. healyi* might be incomplete, and the first four amino acids at the N-terminus might not actually be part of the protein (Figure S1). LBP sequences are highly conserved, with identity/similarity values >80/90% for those of group 2 and 3 species, and around 60/70% between these and those of group 1 species (Table S2).

**Figure 3:**
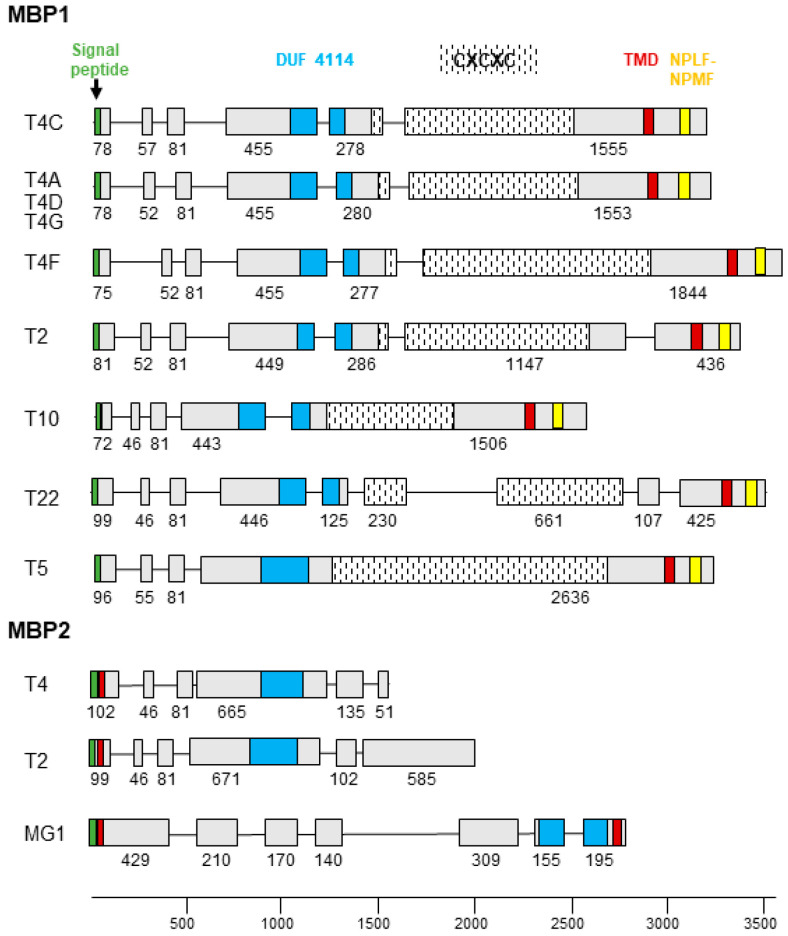
MBP gene structure. Schematic drawing of the exon/intron structure of the MBP1 and MBP2 genes of *Acanthamoeba*. Exons are shown as boxes with the number of nucleotides below, introns as a line. The signal peptide, DUF 4114, CXCXC repeat coding region, transmembrane domain (TMD), and cell signalling NPLF motif are colour coded and shown at the top.

MBP1 is a conventional membrane protein with a signal peptide at the N-terminus and a transmembrane domain located at the C-terminus. The extracellular portion contains a Cys-rich repetitive motif (CXCXC) and a domain of unknown function (DUF 4114), while two NPLF motifs involved in intracellular signalling are located in the intracytoplasmic region [7,9]. MBP1 appears to be specific only to *Acanthamoeba* species of groups 2 and 3, with different gene structure and amino acid sequence depending on the genotype (Table 1) (Figure S2), while shorter MBP-like sequences could be identified in the group 1 species (*A. astronyxis* T7 and *A. byersi* T18), as well as in T4 and T2 genotypes. The resulting protein, labelled MBP2, covers the N-terminal part containing DUF 4114 but lacks the Cys-rich repetitive elements (usually only a single CXCXC motif is present), as well as the intracytoplasmic domain. MBP2 has a signal peptide at the N-terminal followed by a transmembrane motif, although a second short transmembrane motif is predicted at the C-terminus for group 1 species (Figure 3).

It is this short MBP2 which is present in the AHJI01 genome of Neff strain and incorrectly annotated MBP (ACA1_248600; L8GXW7), whereas the true MBP1 is missing, although it is present in the two other Neff genomes. MBP1 sequences from different genotypes are variable, with identity/similarity values <60/75% (Table S3). Moreover, values between MBP1 and MBP2 are even lower (between 25% and 35%), the most conserved part being the DUF 4114 domain (approximately 65% of identical sites).

### 3.3. Molecular Phylogeny of Binding Proteins

**Figure 4:**
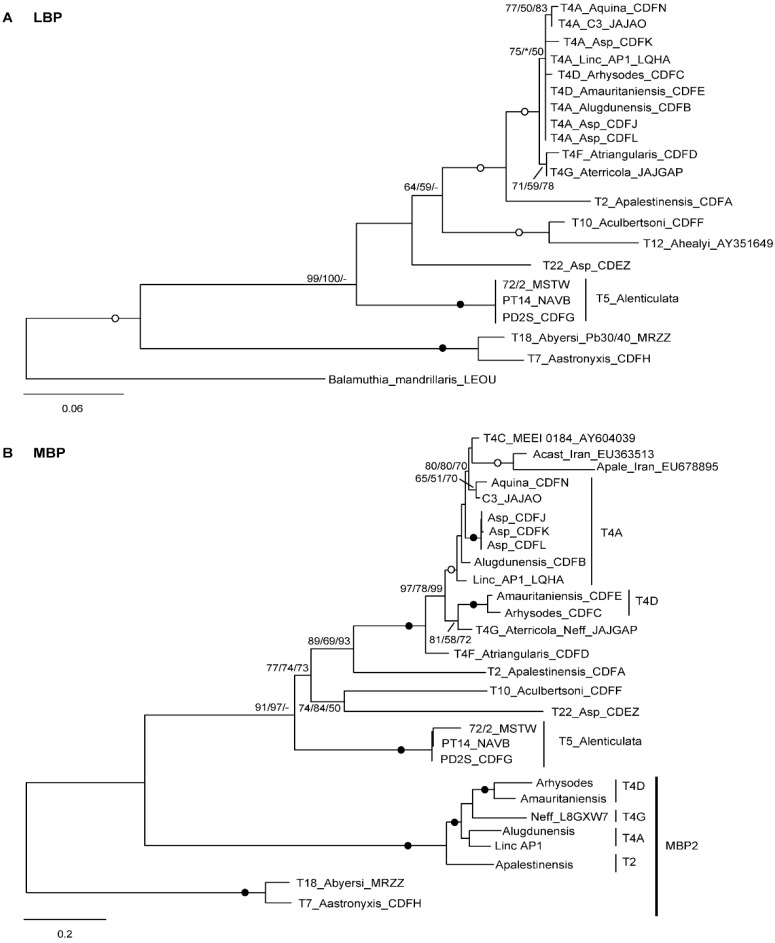
Molecular phylogeny of laminin (**A**) and mannose (**B**) binding proteins. Trees based on amino acid sequences, rooted on the sequence of *Balamuthia mandrillaris* for LBP (**A**), and on MBP2 for MBP (**B**). At the nodes, bootstrap values (1000 replicates) for ML/ME/MP are shown, with filled and open circles for values 100 or >95% with all methods. *, node recovered but support <50%; -, node not recovered.

To assess the evolutionary relationships of LBP and MBP from the different *Acanthamoeba* strains and genotypes, phylogenetic trees were constructed separately for the two proteins from the inferred amino acid sequences (Figure 4).

**Figure 5:**
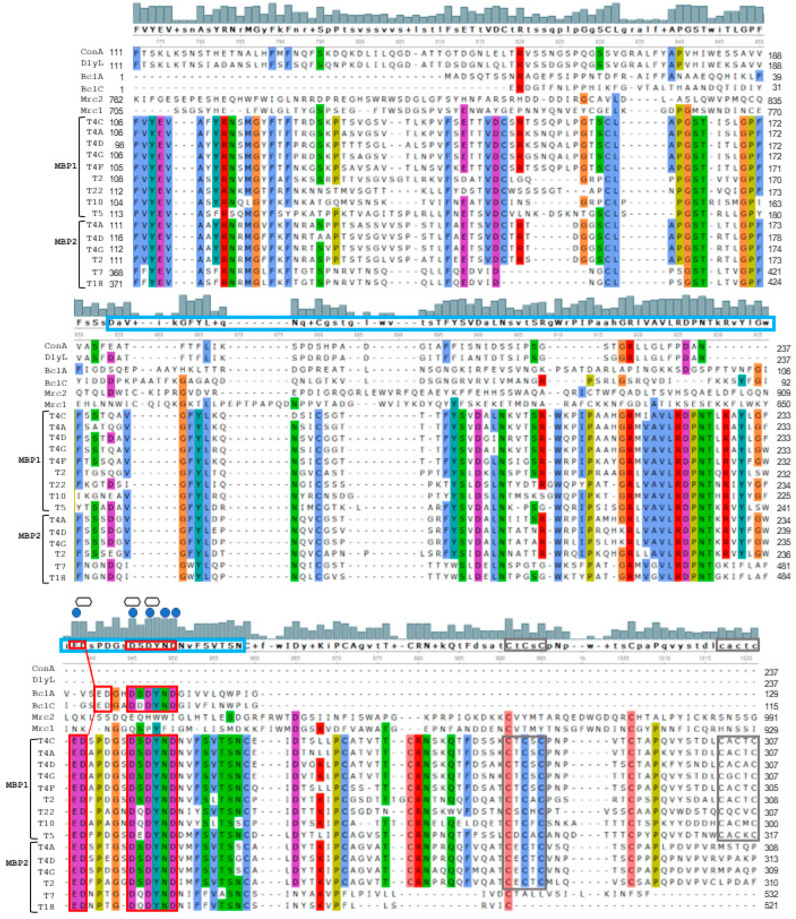
Multiple alignment of *Acanthamoeba* MBP1 and MBP2 with other C-type lectins. Only part of the alignment is shown here, focusing on the DUF 4114 domain, marked with a light blue rectangle in the consensus sequence. The following sequences were used: Concanavalin A (ConA; PDB: 1JBC_A), *Dioclea lasiophylla* lectin (DlyL; Uniprot C0HK27; PDB: 6CJ9_A), BclA (PDB: 2WR9_A) and BclC (PDB: 2XR4_A) lectins from *Burkholderia cenocepacia*, and macrophage receptors Mrc1 (Q61830) and Mrc2 (Q64449). The Bcl ED(xx)GxDxDYND motif, highlighted by a red rectangle, is well conserved in all *Acanthamoeba* MBPs and includes the binding sites for calcium (blue circle) and mannose (hexagon) as shown on consensus sequence histogram. Note that MBP2, except those of group 1 (T7, T18), have a single CXCXC motif corresponding to the first repeat in MBP1 (grey rectangle).

It is noteworthy that the resulting tree topologies almost perfectly mirrors that obtained using the 18S rDNA (Figure 2), with several nodes strongly supported, indicating that the divergences within LBP and MBP occurred by following the diversification of *Acanthamoeba* lineages (Figure 4). This scenario is clearly evident for LBPs, where homologous sequences are available not only for group 1 species (*A. astronyxis* T7 and *A. byersi* T18), but also for *Balamuthia* which can serve as an outgroup (Figure 4A). On the other hand, the picture for MBP is more complicated by the presence of two types of proteins, MBP1 and MBP2, and the absence of a reliable outgroup. However, MBP1 and MBP2 could be homologous, as they show notable sequence conservation in the shared part (see below, Figure 5), and *A. lenticulata* MBP1 clusters with MBP2 in the maximum parsimony tree, albeit with weak support. In any case, while the putative MBP found in group 1 species appears to be the least derived type, MBP1 exhibits a branching pattern consistent with that produced by the 18S rDNA for group 2 and 3 species, spanning down to the T4 subtypes. The same relationships are also recovered for MBP2 from T2 and T4 (Figure 4B).

In the present analysis, MBP1 from strain MEEI (T4C) clusters tightly with MBP1 detected from strain ATCC 30234 which is derived from the strain type of *A. castellanii* ATCC 30011 (T4A). Surprisingly, the MBP1 of *A. castellanii* (Q6J288) is identical to that of MEEI (AAT37864), and the two sequences are even treated as equivalent, i.e., MBP1 of *A. castellanii*, in protein databases. MBP1, as well as MBP2, have been identified in *A. castellanii* ATCC 30234 (T4A) by mass spectrometry [36]. This allowed them to be recognized as such for their overall similarity with MBP1 from MEEI (T4C) and MBP2 (L8GXW7; ACA1_248600) from *A. terricola* (T4G), respectively, but it is unlikely that the sequences of *A. castellanii* could be sequentially identical to the two original proteins. They were therefore excluded from the final analysis.

Another puzzling feature is the branching with MEEI of two partial MBP1 sequences reported as obtained from isolates of *A. castellanii* and *A. palestinensis* (Figure 4B), presumably of genotype T4 and T2, respectively [37,38]. This grouping could be an artefact due to the incompleteness of these sequences, also explaining the relatively long branch. Nevertheless, it is unlikely that either of these sequences could have originated from *A. palestinensis*, and the MBP1 primers developed by the authors have exact matches only in a subset of the T4 strains.

## 4. Discussion

The analysis presented herein reveals that two of the main adhesins of *Acanthamoeba*, MBP and LBP, are constitutive of the amoeba genome, their genes being also present in non-pathogenic strains/species such as *A. terricola* Neff (T4G) (Table 1). The variations observed within the two adhesins, in terms of gene structure and amino acid sequence, appear specific to each *Acanthamoeba* line, reflecting the evolutionary history of the species (Figure 4). This seems congruent when we consider that *Acanthamoeba* is, above all, a free-living amoeba grazing on bacteria, algae, yeasts and small protozoa, and that the different adhesins present on the trophozoite serve primarily to recognize the surface glycoproteins of the prey that will be phagocytized [36,39,40].

High *Acanthamoeba* antibody seroprevalence, even in apparently healthy subjects, suggests wide exposure to the amoeba [41], favoured by its environmental ubiquity. However, not all *Acanthamoeba* strains are capable of infecting and causing disease in humans and other animals. Pathogenic strains are likely those able to adapt to vertebrate tissue which is ultimately an accidental environment, requiring probably various quantitative and qualitative factors, such as the increased expression of adhesins with good affinity for animal tissue glycoproteins, the overproduction of several types of enzymes, or different sensitivities to tissue temperature or osmotic pressure. The pathogenic potential of a strain is in fact often evaluated by plating tests for thermo-tolerance and osmo-tolerance [42,43,44,45], as well as its ability to induce a cytopathic effect (CPE) on cell monolayers [43,45,46], i.e., the successful adhesion and secretion of cytolytic enzymes.

Expression levels of both MBP1 and LBP vary between *Acanthamoeba* strains and correlate with pathogenicity [47,48], as does the diversity of proteases produced. Serine proteases, in particular, have been identified playing a critical role in pathogenicity, such as a 133 kDa mannose-induced protein (MIP-133) which has a cytopathic effect on host cells, and other proteins of different molecular sizes, which attack various components of the extracellular matrix. These proteases are poorly or not expressed in non-pathogenic strains/species [49,50,51,52,53,54].

Group 1 species are of particular interest because they are generally considered non-pathogenic, and therefore used as a negative control. LBP and MBP were not detected [11,55], despite the presence of the corresponding genes, and the inability to induce CPE appears to be due to lack of MIP-133 [50]. However, some data indicate that group 1 species are also potentially pathogenic [56], and furthermore, the evolutionary distance that separates them from other *Acanthamoeba* suggests that some difference might be expected. For example, it is possible that group 1 species use other adhesins to capture their prey since a putative MBP2 and not a true MBP1 could be detected in the analysed genomes. In addition, the total *N*-glycosylation pattern differs from that of group 2 and 3 species [57], implying recognition changes at the molecular level.

Recently, molecular mimicry with *Acanthamoeba* MBP, possibly resulting from convergent evolution, has been reported for mammalian macrophage receptors involved in the antifungal immune response, which recognize mannosylated cell wall proteins of various fungi [58]. Sequence analysis performed herein using more MBP1 and MBP2 sequences confirms affinity with C-type lectin domains, although stronger identity was found with mannose-binding lectins from the opportunistic pathogen *Burkholderia cenocepacia* (Proteobacteria). Notably, the ED(xx)GxDxDYND motif of bacterial lectins involved in calcium and mannose binding in dimeric/trimeric organisation [59,60] is present in the DUF 4114 domain of all *Acanthamoeba* MBP1 and MBP2 (Figure 5).

This suggests that the DUF 4114 domain could participate in the formation of the mannose binding site, possibly by including a divalent cation, in a multimeric complex, which is congruent with a 400 kDa MBP1 composed of three 130 kDa subunits.

The enzymatic treatment of MBP1 from the MEEI strain (T4C) made it possible to estimate that approximately 15% of its apparent mass is due to N-linked oligosaccharides. The de-glycosylated protein shifts from 130 to 110 kDa, the difference with the predicted mass of 85 kDa being probably due to abnormal electrophoretic mobility induced by folding modifications [8]. Interestingly, the 83 kDa MBP1 from *A. culbertsoni* (T10) reported by Kang et al. [61] is congruent not with the predicted molecular mass of the MEEI gene as stated by the authors, but with the fact that the sequence retrieved herein produces a mature protein with a predicted mass of 74.5 kDa having only two *N*-glycosylation sites, compared with six in that of MEEI. Accordingly, the MBP1 from *A. lenticulata* (T5) is expected to have a larger molecular mass given the predicted size of 97.8 kDa (Table 1) and ten *N*-glycosylation sites.

For LBP, the general structure and recognition sites are easily deducible from the conservation of the molecule in all eukaryotes; however, this is not the case for MBP which appears specific to *Acanthamoeba*. It seems that *Acanthamoeba* has indeed developed a new type of lectin to bind the mannose of the surface glycoproteins of the different hunted preys, consisting of the N-terminal region comprising DUF 4114 shared by MBP1 and MBP2, which likely belong to the same protein class. The mannose recognition site would be located in DUF 4114 in all MBPs. The fact that only simpler MBPs seem to be present in group 1 species suggests that the C-terminal part of MBP1, including in particular the CXCXC repeating units, developed in group 2 and 3 species to form a more complex and perhaps more efficient adhesin. The persistence of MBP2 at least in the T4 and T2 strains, and possibly of similar molecules in other genotypes, would indicate the presence of a diversified arsenal of adhesins that may interact with different target glycoproteins.

The presence of genes for two of the main *Acanthamoeba* adhesins, MBP and LBP, in a subset of distinct genotypes suggests their ubiquity within the genus. It is likely that both MBP and LBP serve primarily as ligands to catch microbial prey in the environment and only incidentally as virulence factors by recognizing glycoproteins from animal tissues. The observed variations are largely consistent with the branching of 18S lineages, indicating that the changes within the two adhesins are mainly due to speciation within *Acanthamoeba*. It is obviously essential to insist on the importance of properly recognizing the different lineages of *Acanthamoeba*, in order to better appreciate these differences and also to explain certain previous conflicting results, while avoiding persistent confusion on misattributed strains.

## Figures and Tables

**Table 1:** Summary of *Acanthamoeba* binding proteins data.

GT	Species	Strain	Sequence Source ^1^	Mannose Binding Protein				Laminin Binding Protein			
				Gene		Protein		Gene		Protein	
				nt ^2^	exons	aa	kDa ^3^	nt ^2^	exons	aa	kDa ^3^
T4A	*A. quina*	Vil3	CDFN01	3104	6	833	85.2	1084	3	265	29.3
T4A	*Acanthamoeba* sp.	undet.	CDFL01	3106	6	833	85.3	1090	3	266	29.3
T4A	*Acanthamoeba* sp.	undet.	CDFJ01	3105	6	833	85.4	1090	3	266	29.3
T4A	*Acanthamoeba* sp.	undet.	CDFK01	3120	6	833	85.5	1092	3	248	27.2
T4A	*A. lugdunensis*	L3a	CDFB01	3112	6	833	85.1	1085	3	266	29.3
T4A	*Acanthamoeba* sp.	C3	JAJGAO01	3151	6	833	85.2	1082	3	266	29.4
T4A	*Acanthamoeba* sp.	Linc-AP1	LQHA01	3118	6	833	85.0	1085	3	266	29.3
T4C	*Acanthamoeba* sp.	MEEI 0184	AY604039 ^1^	3156	6	833	85.2	not available			
T4D	*A. rhysodes*	Singh	CDFC01	3168	6	833	85.1	1066	3	266	29.3
T4D	*A. mauritaniensis*	1652	CDFE01	3218	6	834	84.9	1077	3	266	29.3
T4G	*A. terricola*	Neff	JAJGAP01	3159	6	834	85.1	1082	3	264	29.1
T4G	*A. terricola*	Neff	AEYA01	3159	6	834	85.1	1082	3	264	29.1
T4G	*A. terricola*	Neff	AHJI01	no results ^4^				1082	3	264	29.1
T4F	*A. triangularis*	SH621	CDFD01	3507	6	928	95.0	1063	3	265	29.3
T2	*A. palestinensis*	Reich	CDFA01	3365	7	844	87.0	1043	2	265	29.4
T10	*A. culbertsoni*	Lilly A1	CDFF01	2520	5	716	74.5	1100	3	260	28.8
T12	*A. healyi*	OC-3A	AY351649 ^1^	not available				771	1	252	28.3
T22	*Acanthamoeba* sp.	undet.	CDEZ01	3440	9	747	76.4	967	2	260	29.5
T5	*A. lenticulata*	PD2S	CDFG01	3138	4	956	97.7	967	2	260	28.9
T5	*A. lenticulata*	72/2	MSTW01	3152	4	956	97.8	967	2	260	28.9
T5	*A. lenticulata*	PT14	NAVB01	3150	4	956	97.8	967	2	260	28.9
T7	*A. astronyxis*	undet.	CDFI01	no results ^4^				1071	4	233	25.9
T7	*A. astronyxis*	R&H	CDFH01	no results ^4^				1071	4	233	25.9
T18	*A. byersi*	Pb30/40	MRZZ01	no results ^4^				1072	4	232	25.9
*Balamuthia mandrillaris*		2046	LEOU01	no results				871	2	264	28.8

^1^ Sequences from T4C and T12 are the original query sequences for MBP1 and LBP; ^2^ Length spanning start/stop codons; ^3^ Predicted molecular weight of non-glycosylated mature protein; ^4^ Analysis recovered MBP2.

## Data Availability

The data presented in this study are available in Supplementary Material.

## Supplementary Materials

The following supporting information can be downloaded at: https://www.mdpi.com/article/10.3390/microorganisms10112162/s1, Figure S1: Multiple alignment of full-length laminin-binding proteins (LBPs) from *Acanthamoeba* and *Balamuthia*; Figure S2: Multiple alignment of *Acanthamoeba* full-length mannose-binding proteins (MBP1); Table S1: List of *Acanthamoeba* binding proteins accession numbers; Table S2: Identity and similarity percentage values for LBP; Table S3: Identity and similarity percentage values for MBP.
