# Supplementary material for: Acanthamoeba Mannose and Laminin Binding Proteins Variation across Species and Genotypes
Source: Microorganisms. 2022 Oct 31;10(11):2162. doi: 10.3390/microorganisms10112162 (PMC9692275; doi:10.3390/microorganisms10112162)

Figure S1. Multiple alignment of full-length laminin-binding proteins (LBPs) from *Acanthamoeba* and *Balamuthia*. The transmembrane domain (TMD) and the three putative laminin binding sites, the palindromic LMWWML (LMFWLL), the Direct Binding Region (DBR), and TEDWS (TEEWG), are highlighted by coloured rectangles on the consensus sequence. Note for *A. healyi* the discrepancy at both ends, highlighted by a red rectangle, suggesting possible sequence incompleteness at the C-terminus.

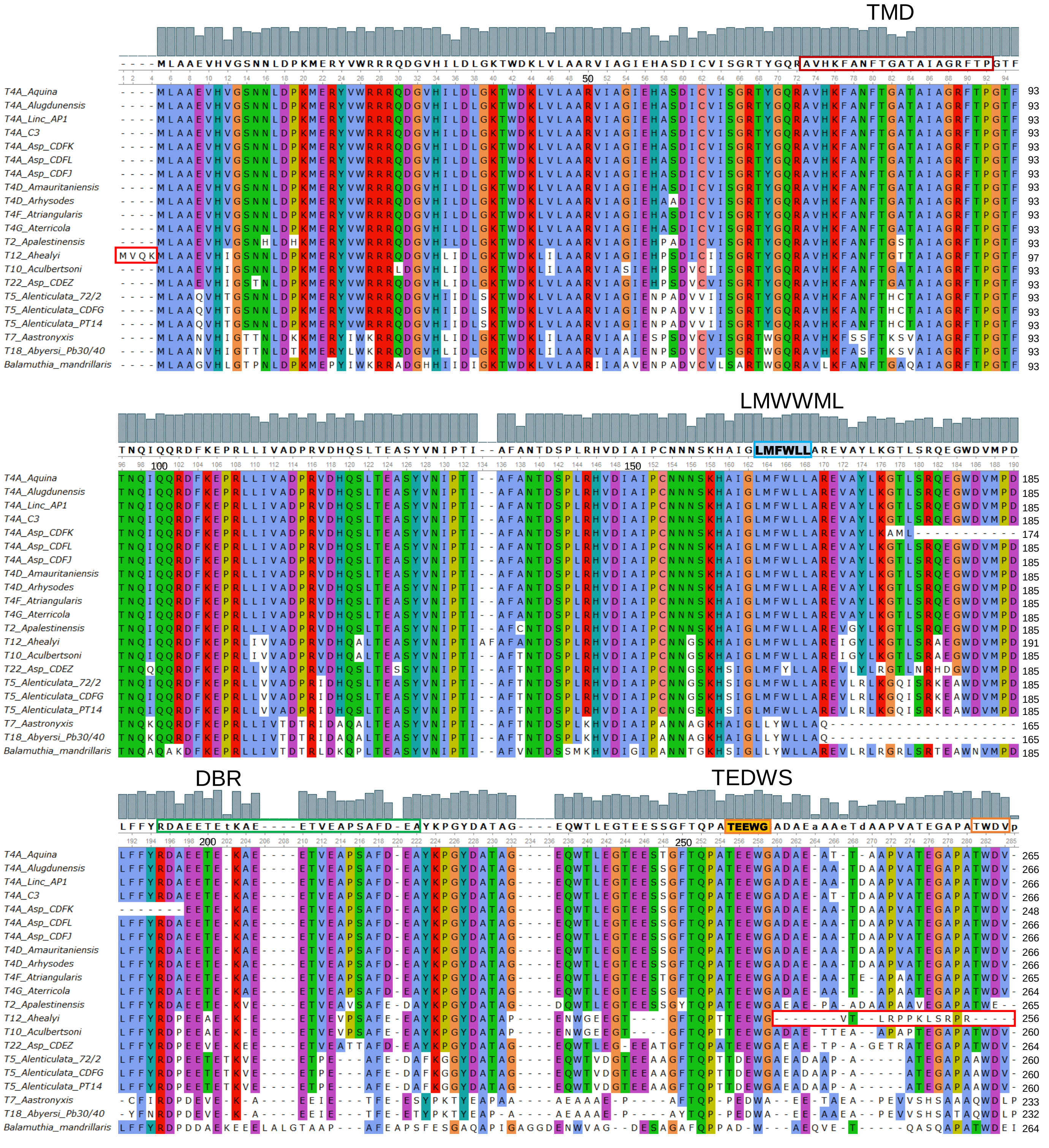

Supplement: Supplementary file 1 [file microorganisms-10-02162-s001.zip › Figure S1.pdf]
