# Supplementary material for: Acanthamoeba Mannose and Laminin Binding Proteins Variation across Species and Genotypes
Source: Microorganisms. 2022 Oct 31;10(11):2162. doi: 10.3390/microorganisms10112162 (PMC9692275; doi:10.3390/microorganisms10112162)
Supplement: Supplementary file 1 [file microorganisms-10-02162-s001.zip › Figure S2.pdf]

Figure S2. Multiple alignment of *Acanthamoeba* full-length mannose-binding proteins (MBP1). The DUF 4114 region, the CXCXC motifs, the transmembrane domain (TMD) and the NPLF motif are highlighted by colored rectangles on the consensus sequence. Note that some sequences present either additional motifs (*A. lenticulata* T5, *A. triangularis* T4F) or extended gaps (*A. culbertsoni* T10, *Acanthamoeba* sp. T22) which seem to be specific to each line.

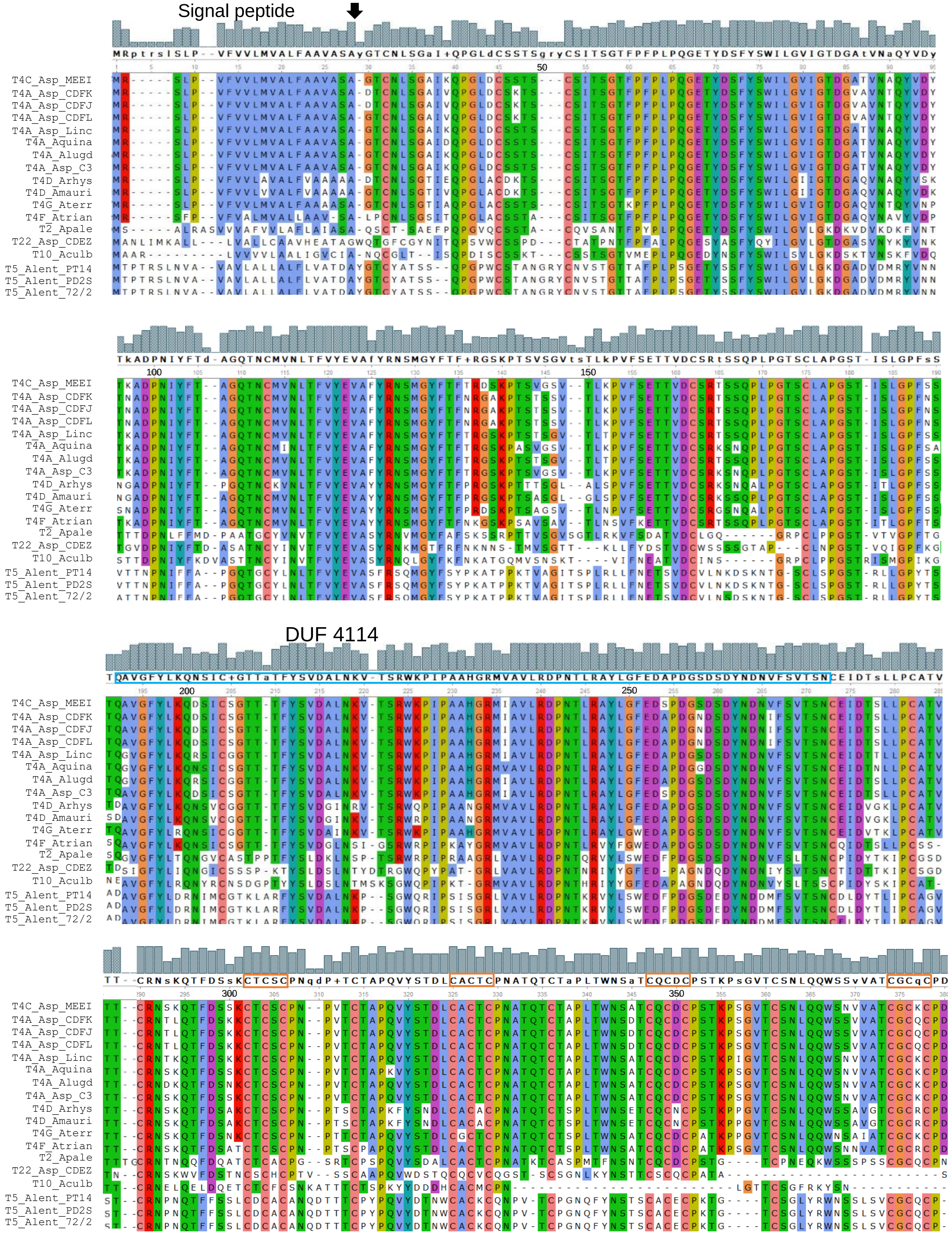

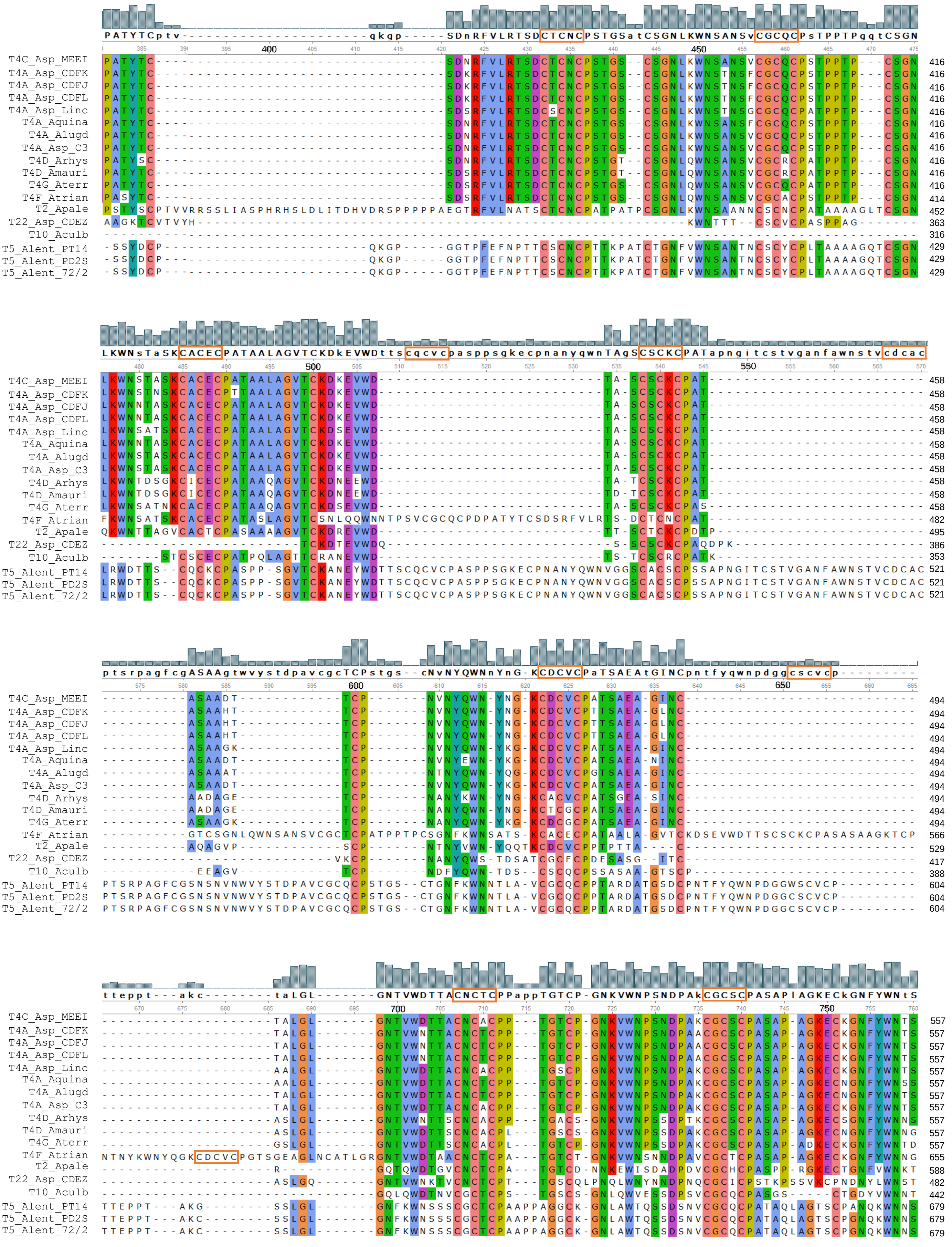
T4C_Asp_MEEI
T4A_Asp_CDFK
T4A_Asp_CDFJ
T4A_Asp_CDFL
T4A_Asp_Linc
T4A_Aquina
T4A_Alugd
T4A_Asp_C3
T4D_Arhys
T4D_Amauri
T4G_Aterr
T4F_Atrian
T2_Apale
T22_Asp_CDEZ
T10_Aculb
T5_Alent_PT14
T5_Alent_PD2S
T5_Alent_72/2

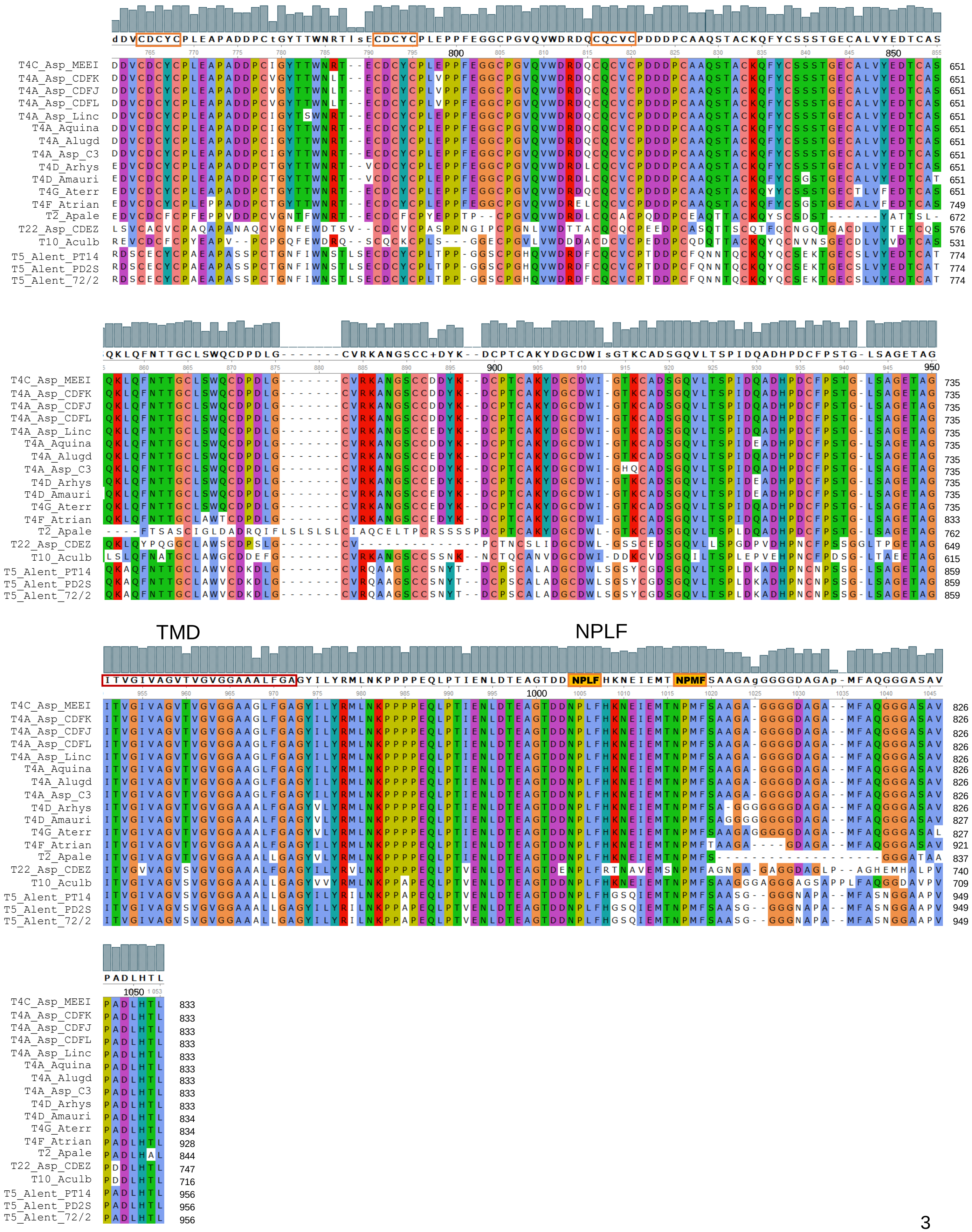
TMD
NPLF
