# Supplementary material for: Acanthamoeba Mannose and Laminin Binding Proteins Variation across Species and Genotypes
Source: Microorganisms. 2022 Oct 31;10(11):2162. doi: 10.3390/microorganisms10112162 (PMC9692275; doi:10.3390/microorganisms10112162)
Supplement: Supplementary file 1 [file microorganisms-10-02162-s001.zip › Table S1.pdf]

Table S1. List of *Acanthamoeba* binding proteins accession numbers.

GT	species	strain	WSG project	accession nos. [1]	
				**Mannose Binding Protein MBP1**	**Laminin Binding Protein LBP** [2]
T4A	*A. quina*	Vil3	CDFN01	CDFN01055557:12308-15411	CDFN01057992:c1259-176
T4A	*Acanthamoeba* sp.	undet.	CDFL01	CDFL01207565:c524-614; CDFL01208350: 4453-7482; CDFL01217467:c1332-1451; CDFL01205628.1:c1016-417	CDFL01212578:c1122-33
T4A	*Acanthamoeba* sp.	undet.	CDFJ01	CDFJ01222928:4349-7350; CDFJ01191546:498-600	CDFJ01208995:c1122-33
T4A	*Acanthamoeba* sp.	undet.	CDFK01	CDFK01178816:c479-500; CDFK01196080:c1082-1; CDFK01219318:c3000-1; CDFK01110294 1-177; CDFK01213929: c5869-5267	CDFK01218873:2507-3597
T4A	*A. lugdunensis*	L3a	CDFB01	CDFB01056783:c835-1; CDFB01056784:1-1277; CDFB01057839:1-1823; CDFB01049883:c3360-2409	CDFB01065890:5579-6663
T4A	*Acanthamoeba* sp.	C3	JAJGAO01	JAJGAO010000003:c190347-187196	JAJGAO010000024:c303058-301977
T4A	*Acanthamoeba* sp.	Linc-AP1	LQHA01	LQHA01002101:1564-4682	LQHA01000768:c5284-4200
T4D	*A. rhysodes*	Singh	CDFC01	CDFC01051249:c1326-1; CDFC01060751:c456-1; CDFC01043110:c2212-776	CDFC01062378:c2707-1642
T4D	*A. mauritaniensis*	1652	CDFE01	CDFE01049898:2257-5474	CDFE01024373:c98-1; CDFE01012671:c127-1; CDFE01020295:c127-1; CDFE01049673:c2767-1854
T4G	*A. terricola*	Neff	JAJGAP01	JAJGAP010000003:1659702-1662860	JAJGAP010000022:c306652-305571
T4G	*A. terricola*	Neff	AEYA01	AEYA01002376:202059-205217	AEYA01001381:c3313-2232
T4G	*A. terricola*	Neff	AHJI01	no result [3]	NW_004457598:c175024-176105 ACA1_385450 (putative LBP, pseudo)
T4F	*A. triangularis*	SH621	CDFD01	CDFD01052511:2071-3639; CDFD01027228:c2092-1; CDFD01027227:1-1209	CDFD01037165:18655-19717
T2	*A. palestinensis*	Reich	CDFA01	CDFA01025191:14111-17415	CDFA01025720:86346-87388
T10	*A. culbertsoni*	Lilly A1	CDFF01	CDFF01071382:16785-19304	CDFF01068896:342-1441
T22	*Acanthamoeba* sp.	undet.	CDEZ01	CDEZ01024055:2187-5626	CDEZ01023550:c354-1; CDEZ01018663:c8501-7358
T5	*A. lenticulata*	PD2S	CDFG01	CDFG01072604:283-3420	CDFG01076243:c20135-19169
T5	*A. lenticulata*	72/2	MSTW01	MSTW01007909:1630-3768; MSTW01007910:3-1085	MSTW01004628:c4634-3668
T5	*A. lenticulata*	PT14	NAVB01	NAVB01001349:c3792-643	NAVB01006311:c3066-2100
T7	*A. astronyxis*	undet.	CDFI01	no result [3]	CDFI01105374:10207-11277
T7	*A. astronyxis*	R&H	CDFH01	no result [3]	CDFH01091946:266-1336
T18	*A. byersi*	Pb30/40	MRZZ01	no result [3]	MRZZ01011069:c2240-1130

[1] The range indicates start/stop codons. Multiple entries indicate overlapping contings giving the complete gene sequence.

[2] Many genomes have multiple (two to four) identical LBP gene copies, not reported here for simplicity.

[3] The analysis gave MBP2.
