# Supplementary material for: Acanthamoeba Mannose and Laminin Binding Proteins Variation across Species and Genotypes
Source: Microorganisms. 2022 Oct 31;10(11):2162. doi: 10.3390/microorganisms10112162 (PMC9692275; doi:10.3390/microorganisms10112162)
Supplement: Supplementary file 1 [file microorganisms-10-02162-s001.zip › Table S2 Table S3.pdf]

Table S2. Identity (top right, blue) and similarity (bottom left, green) values (%) for Laminin-Binding Proteins (LBP)

	GT	species	1	2	3	4	5	6	7	8	9	10	11	12	13	14	15	16	17	18
1	T4A	*A. quina*		98.9	98.9	99.2	91.4	98.9	98.9	98.5	98.9	98.5	91.7	82.0	87.2	87.5	82.3	61.0	60.5	61.5
2	T4A	*A. lugdunensis*	99.2		100	99.6	92.5	100	100	99.6	98.5	98.9	92.5	80.6	86.5	87.3	82.4	60.3	60.7	61.3
3	T4A	Asp Linc_AP1	99.2	100		99.6	92.5	100	100	99.6	98.5	98.9	92.5	80.6	86.5	87.3	82.4	60.3	60.7	61.3
4	T4A	Asp C3	99.6	99.6	99.6		92.1	99.6	99.6	99.2	98.1	98.5	92.1	80.6	86.8	87.2	82.4	60.7	60.3	61.3
5	T4A	Asp CDFK	92.1	92.5	92.5	92.1		92.5	92.5	92.1	91.0	91.4	85.0	73.9	79.7	80.8	76.4	63.5	64.3	56.2
6	T4A	Asp CDFL	99.2	100	100	99.6	92.5		100	99.6	98.5	98.9	92.5	80.6	86.5	87.3	82.4	60.3	60.7	61.3
7	T4D	*A. mauritaniensis*	99.2	100	100	99.6	92.5	100		99.6	98.5	98.9	92.5	80.6	86.5	87.3	82.4	60.3	60.7	61.3
8	T4D	*A. rhysodes*	99.2	100	100	99.6	92.5	100	100		98.1	98.5	92.9	80.2	86.1	86.9	82.8	59.9	60.3	61.7
9	T4F	*A. triangularis*	98.9	99.2	99.2	98.9	92.1	99.2	99.2	99.2		99.2	92.1	81.6	86.8	87.5	82.7	60.7	61.0	61.5
10	T4G	*A. terricola*	98.9	98.9	98.9	98.5	92.0	98.9	98.9	98.9	99.6		92.1	81.6	86.4	87.5	83.0	60.5	60.9	61.0
11	T2	*A. palestinensis*	95.8	95.9	95.9	95.5	88.7	95.9	95.9	95.9	96.2	95.8		79.4	83.1	83.9	82.4	58.8	60.3	60.8
12	T12	*A. healyi*	86.8	85.3	85.3	85.3	82.9	85.3	85.3	85.3	86.4	86.4	86.0		90.1	80.1	76.0	60.5	60.9	58.8
13	T10	*A. culbertsoni*	91.3	91.0	91.0	91.4	86.2	91.0	91.0	91.0	90.9	90.9	90.6	91.2		85.6	80.7	63.2	63.2	61.3
14	T22	Asp CDEZ	93.2	93.6	93.6	93.2	86.4	93.6	93.6	93.6	93.2	93.9	93.6	86.4	90.9		82.0	62.0	63.2	62.5
15	T5	*A. lenticulata* [a]	90.2	90.2	90.2	90.2	85.8	90.2	90.2	90.2	90.6	91.3	90.6	83.8	88.8	90.2		60.7	61.8	63.0
16	T7	*A. astronyxis*	71.7	71.1	71.1	71.4	76.2	71.1	71.1	71.1	71.3	71.2	70.2	71.8	72.3	73.1	71.2		95.3	59.3
17	T18	*A. byersi*	70.9	71.4	71.4	71.1	75.8	71.4	71.4	71.4	71.7	71.6	71.3	72.2	73.1	74.2	71.5	97.4		58.5
18	-	*Balamuthia*	75.8	75.6	75.6	75.6	69.7	75.6	75.6	75.6	75.8	75.4	75.8	73.5	77.3	78.4	75.4	68.9	69.3	

[a] The three strains of *A. lenticulata* have identical sequences

Table S3. Identity (top right, blue) and similarity (bottom left, green) values (%) for *Acanthamoeba* Mannose-Binding Proteins 1 (MBP1)

	GT	species	1	2	3	4	5	6	7	8	9	10	11	12	13	14	15	16	17	18
1	T4C	Asp MEEI_0184		95.7	95.8	95.9	96.6	97.1	97.8	99.3	87.5	88.2	91.7	78.7	59.3	50.4	51.8	51.8	51.6	51.8
2	T4A	Asp CDFK	97.0		99.6	99.5	94.5	94.0	95.2	95.2	86.3	86.7	89.4	77.4	58.8	50.4	51.7	51.1	51.3	51.5
3	T4A	Asp CDFJ	97.2	99.8		99.9	94.5	94.4	95.3	95.3	86.4	86.8	89.4	77.6	59.1	50.4	52.0	51.2	51.4	51.6
4	T4A	Asp CDFL	97.4	99.6	99.9		94.6	94.5	95.4	95.4	86.3	86.9	89.6	77.6	59.1	50.7	52.0	51.3	51.5	51.7
5	T4A	Asp Linc_AP1	97.7	96.3	96.4	96.5		95.3	97.4	96.4	87.8	88.7	92.0	78.8	59.3	50.9	51.7	51.9	51.7	51.9
6	T4A	*A. quina*	98.6	96.4	96.6	96.8	97.2		96.4	97.4	87.6	88.0	90.5	78.1	59.1	50.8	52.2	51.0	51.1	51.3
7	T4A	*A. lugdunensis*	98.2	96.4	96.6	96.8	98.2	98.0		97.5	87.8	88.5	91.2	79.0	59.8	50.5	51.7	51.2	51.5	51.7
8	T4A	Asp C3	99.5	96.8	97.0	97.1	97.7	98.8	98.2		87.5	88.0	91.6	78.3	58.9	50.3	51.8	51.8	51.6	51.8
9	T4D	*A. rhysodes*	92.4	91.5	91.5	91.4	93.0	92.3	92.4	92.4		96.2	89.2	75.6	58.7	49.8	51.5	52.1	52.2	52.4
10	T4D	*A. mauritaniensis*	92.8	91.7	91.7	91.8	93.2	92.4	92.7	92.8	97.7		90.4	76.4	58.3	50.7	52.1	51.7	51.9	52.1
11	T4G	*A. terricola*	95.4	93.6	93.8	93.9	95.3	94.5	94.6	95.3	93.8	94.7		77.9	59.3	49.8	52.0	52.4	52.6	52.8
12	T4F	*A. triangularis*	83.7	82.5	82.9	82.9	83.8	83.8	83.5	83.7	81.5	82.4	83.4		58.0	46.4	45.9	53.7	54.0	54.1
13	T2	*A. palestinensis*	73.6	72.9	73.0	73.0	73.1	73.5	73.6	73.6	72.5	71.3	72.6	68.3		46.1	46.6	47.2	47.2	47.1
14	T22	Asp CDEZ	64.3	63.3	63.3	63.6	63.5	63.4	63.5	63.9	62.7	63.2	63.3	58.0	60.0		53.5	41.9	42.0	42.1
15	T10	*A. culbertsoni*	63.0	63.3	63.5	63.4	63.1	62.9	62.8	63.0	63.1	63.5	62.9	57.0	60.0	67.5		43.1	43.2	43.2
16	T5	*A. lenticulata* 72/2	64.9	63.7	63.7	63.8	64.6	64.2	63.8	64.9	64.7	64.5	65.1	68.3	59.2	54.3	54.6		99.7	99.4
17	T5	*A. lenticulata* PT14	64.4	63.7	63.7	63.8	64.1	64.3	64.0	64.3	64.9	64.5	64.3	68.6	58.9	54.6	54.7	99.7		99.7
18	T5	*A. lenticulata* CDFG	63.8	63.8	63.8	63.9	64.2	64.4	64.1	64.4	65.0	65.3	64.5	68.8	58.8	54.7	54.8	99.5	99.8	
